# Repurposing Molnupiravir for COVID-19: The Mechanisms of Antiviral Activity

**DOI:** 10.3390/v14061345

**Published:** 2022-06-20

**Authors:** Ashley Jia Wen Yip, Zheng Yao Low, Vincent T. K. Chow, Sunil K. Lal

**Affiliations:** 1 School of Science, Monash University, Bandar Sunway, Subang Jaya 47500, Selangor Darul Ehsan, Malaysia; ashley.yip@monash.edu (A.J.W.Y.); zheng.low@monash.edu (Z.Y.L.); 2 Infectious Diseases Translational Research Program, Department of Microbiology and Immunology, Yong Loo Lin School of Medicine, National University of Singapore, Singapore 117545, Singapore; micctk@nus.edu.sg; 3 Tropical Medicine & Biology Platform, Monash University, Subang Jaya 47500, Selangor Darul Ehsan, Malaysia

**Keywords:** COVID-19, SARS-CoV-2, repurposing, repositioning, antiviral, Molnupiravir, NHC, N4-hydroxycytidine

## Abstract

Molnupiravir is a β-d-N4-hydroxycytidine-5′-isopropyl ester (NHC) compound that exerts antiviral activity against various RNA viruses such as influenza, SARS, and Ebola viruses. Thus, the repurposing of Molnupiravir has gained significant attention for combatting infection with SARS-CoV-2, the etiological agent of COVID-19. Recently, Molnupiravir was granted authorization for the treatment of mild-to-moderate COVID-19 in adults. Findings from in vitro experiments, in vivo studies and clinical trials reveal that Molnupiravir is effective against SARS-CoV-2 by inducing viral RNA mutagenesis, thereby giving rise to mutated complementary RNA strands that generate non-functional viruses. To date, the data collectively suggest that Molnupiravir possesses promising antiviral activity as well as favorable prophylactic efficacy, attributed to its effective mutagenic property of disrupting viral replication. This review discusses the mechanisms of action of Molnupiravir and highlights its clinical utility by disabling SARS-CoV-2 replication, thereby ameliorating COVID-19 severity. Despite relatively few short-term adverse effects thus far, further detailed clinical studies and long-term pharmacovigilance are needed in view of its mutagenic effects.

## 1. Introduction

The recent COVID-19 pandemic has exerted a significant impact on global health and the economy. The etiological agent causing this disastrous pandemic is known as the Severe Acute Respiratory Syndrome Coronavirus-2 (SARS-CoV-2). The term “coronavirus” is no stranger to many as the emergence of coronaviruses causing SARS and Middle East Respiratory Syndrome (MERS) have left an indelible impression over the past two decades. Afflicting more than 530 million individuals accompanied by 6.3 million deaths worldwide, SARS-CoV-2 infection continues to progress at a very rapid rate on a global scale [1]. The zoonotic origin of SARS-CoV-2 has been postulated to be bats, similar to its previous counterpart, SARS-CoV [2]. Despite exacting a lower estimated case fatality rate (3.4%) compared to its predecessors (9.6% for SARS-CoV, and 40% for MERS-CoV), SARS-CoV-2 shows greater transmissibility along with the rapid emergence of new variants [3]. To facilitate public health and research activities, WHO has reclassified the variants of SARS-CoV-2—there are variants of concern (VOC), variants of interest (VOI), and variants under monitoring (VUM), which are named after the Greek alphabets [4]. The more recent VOCs are delta and omicron with Pango lineages B.1.617.2 from India, and B.1.1.529 first detected in South Africa, respectively [4]. On the other hand, VOIs fall under the “previous circulating” category, encompassing the epsilon, zeta, iota, eta, theta, kappa, lambda, and mu variants [4].

It is noteworthy that COVID-19 may lead to certain critical complications, such as pneumonia, septic shock, multiple organ failure, and the more pronounced acute respiratory distress syndrome or ARDS [5]. In such a time of urgent need, drug repositioning attempts represent a suitable strategy to develop antiviral therapy against COVID-19. Drug repositioning—also known as drug repurposing, drug recycling, or drug reprofiling—is described as an alternative approach to find new uses for a previously established drug to treat disease(s) other than its initially intended one [6]. The benefits of drug repositioning undoubtedly include lower costs since the drug has already undergone rigorous safety and pharmacokinetic profiling, with a shorter drug development time for its new repositioned target [7]. Currently, there is an approved nucleoside analog-based antiviral agent against COVID-19, i.e., Remdesivir [8]. A number of existing drug candidates have been proposed and/or evaluated as antivirals against SARS-CoV-2, e.g., Lopinavir-Ritonavir, Ivermectin, and others in the pipeline [9]. 

Lopinavir-Ritonavir is one of the first co-formulated HIV-1 protease inhibitors [10]. It was reported to have brought substantial improvements over the previous standard therapy with nucleoside reverse transcriptase inhibitors (NRTIs) [11]. The combination of Lopinavir-Ritonavir has been investigated for its efficacy for COVID-19 treatment, in view of its formation of stable complexes with viral 3-chymotrypsin-like protease (3CLpro) or main protease (Mpro) which regulate the proteolytic activity for viral replication [12]. Ivermectin, an anti-parasitic drug, has gained attention as a potential repurposed drug against COVID-19. It was first developed and commercialized as a veterinary drug in 1981 due to its promising nematicidal, acaricidal, and insecticidal activities [13]. Ivermectin is an FDA-approved drug for treating onchocerciasis and *Strongyloides* infection in humans [14,15]. The proposed modes of SARS-CoV-2 inhibition by Ivermectin include: the disruption of host importin heterodimer complex (IMPα/β1), inhibition of viral entry via the host angiotensin-converting enzyme 2 (ACE2) receptor, and disruption of the viral 3CLpro enzyme—thereby reducing the efficiency of viral replication [15]. 

Notably, RNA-dependent RNA polymerase (RdRp) represents a preferred target for developing drug inhibitors against RNA viruses, and SARS-CoV-2 is no exception. This is attributed to the highly conserved nature of RdRp domains within the *Coronaviridae* family, especially with respect to the predecessor SARS-CoV. Comparative genomic analysis reveals high homology of RdRp domains between SARS-CoV and SARS-CoV-2 with 96.3%, 98.8%, and 97.5% similarity in NSP12, NSP7, and NSP8, respectively—rendering RdRp a highly suitable target for drug repositioning [16,17,18]. In particular, Remdesivir, a broad-spectrum nucleoside analog inhibitor of RdRp, was the first intravenous antiviral drug against COVID-19 approved by the US Food and Drug Administration (FDA) [19]. Although initially developed against Ebola virus, Remdesivir also exhibits positive antiviral activity against multiple viruses, such as filoviruses, paramyxoviruses, coronaviruses (e.g., SARS-CoV), and others [20,21]. Given the favorable clinical trial outcomes coupled with the close RdRp similarity between SARS-CoV-2 and SARS-CoV, this therapeutic agent has been approved for the management of severe COVID-19 patients. 

More recently, Molnupiravir, a newly emerged repositioned synthetic nucleoside-derived RdRp inhibitor, has also gained attention for the treatment of COVID-19. This drug is also known as β-d-N4-hydroxycytidine-5′-isopropyl ester (NHC) or Emory Institute of Drug Development-2801 (EIDD-2801) [22]. The first synthesis of Molnupiravir was reported by the Drug Innovation Ventures at Emory University back in 2018—later acquired by Ridgeback Biotherapeutics and partnered with Merck for further development [23,24]. Molnupiravir has been proven effective in multiple antiviral treatments against influenza A virus (IAV), Venezuelan equine encephalitis virus (VEEV), Ebola virus, SARS-CoV, and most recently SARS-CoV-2 [25,26]. Remdesivir acts as a nucleoside analog that interferes directly with RdRp activity. However, the mechanism of Molnupiravir is via its interactions with the RNA building blocks instead. Molnupiravir works primarily as a mutagenesis agent that induces RNA mutations by incorporating the incorrect nucleo-base into the viral RNA genome leading to catastrophic errors [27]. The RdRp then replicates and generates new virions with the errors brought forward, thereby effectively hampering viral replication capacity of the new virions [27].

The UK Medicines and Healthcare Products Regulatory Agency recently approved Molnupiravir as an oral antiviral drug against COVID-19 for adults in emergency and urgent settings [28]. This agent has the potential to ameliorate disease severity and contribute to lower morbidity and mortality among COVID-19 patients. Hence, this review aims to highlight consolidated knowledge on the molecular mechanisms underpinning the inhibition of SARS-CoV-2 replication of by Molnupiravir.

## 2. Antiviral Action of Molnupiravir

Previous studies have revealed the efficacy of Molnupiravir against multiple virus infections. Given that viral RdRp plays a substantial role in many RNA viruses, it is of interest to unravel the mechanisms of action of Molnupiravir against SARS-CoV-2. In view of the relative conservation of RdRp in coronaviruses, especially between SARS-CoV and SARS-CoV-2, it is worthwhile to describe the antiviral mechanisms of Molnupiravir in the context of the SARS-CoV-2 replication cycle.

### 2.1. Influenza A Virus (IAV)

The influenza A virus is a negative-sense RNA virus that consists of eight unique RNA segments that encode 14 known proteins [29]. To produce new virions, IAV needs to undergo a series of genomic replication processes driven by RNA polymerase enzymes, known as RdRp. Being a negative-sense RNA virus, the RdRp synthesizes numerous complementary positive-strand RNAs which serve as templates to generate more negative-sense RNA viral genomes for viral replication. In addition, the negative-strand RNAs also serve as templates to manufacture more positive-strand RNAs that constitute the mRNAs to encode functional viral proteins [30]. In this regard, RdRp incorporates new nucleotide triphosphates (NTPs) into the newly synthesized complementary strands. As a mutagen, NHC permits incorporation of cytidine into the viral RNA genome, leading to increased frequency of tautomeric interconversions of C-to-U and G-to-A, thereby culminating in catastrophic errors in the newly produced virions [31]. In vivo studies have also provided evidence to support the efficacy of NHC against IAV [31,32]. Excess levels of exogenous cytidine or uridine indicate that NHC is recognized as a pyrimidine analog by IAV. This results in significantly reduced IAV replication in the presence of NHC, accompanied by a greater frequency of C-to-U, G-to-A, and A-to-G mutations at 400 mg/kg dosage [33]. These findings suggest the potential of Molnupiravir as an effective antiviral against IAV. 

### 2.2. Venezuelan Equine Encephalitis Virus (VEEV)

The positive-sense single-stranded RNA Venezuelan equine encephalitis virus belongs to the genus of New World alphaviruses. VEEV shares the trait of mosquito-borne transmission with its counterparts, i.e., Chikungunya virus (CHIKV), Eastern equine encephalitis virus (EEEV), and Western equine encephalitis virus (WEEV) [34]. Being an encephalitis virus that targets the central nervous system, antiviral drugs need to penetrate the blood-brain barrier to be effective against VEEV [35,36]. NHC induces a high rate of mutations in VEEV genomic RNA, where 2 μM drug concentration leads to at least a ten-fold increase in new mutations, with U-to-C or C-to-U transitions being the most prevalent. It was demonstrated that most VEEV released from the NHC-treated cells contained mutated viral genomes that were incapable of replicating. Moreover, VEEV mutants remained highly sensitive to NHC despite 20 passages [36]. In addition, 90% of mice survived lethal intranasal VEEV challenge when treated with 500 mg/kg and 300 mg/kg doses (twice daily, b.i.d.), with an 80% survival rate upon administration of 150 mg/kg dose (b.i.d.)—compared to the non-NHC control group of mice that died after 6 days. Notably, virus titers were undetectable or very low in the brain samples of all NHC-treated mice [37]. In light of this, Molnupiravir shows great promise for prophylactic and antiviral therapies. 

### 2.3. SARS-CoV and SARS-CoV-2

Both SARS-CoV and SARS-CoV-2 are positive-sense, single-stranded RNA viruses that contain approximately 30,000 nucleotides [38]. Given the high genetic identity of RdRp between SARS-CoV-2 and SARS-CoV (99.4% sequence similarity and 96.4% sequence identity), it is crucial to understand the mode of inhibition of RdRp in SARS-CoV as well as SARS-CoV-2 [39]. The mode of action of NHC against SARS-CoV RdRp is no different from its other counterparts, and several in vivo studies have proven the efficacy of NHC against multiple coronaviruses [40,41]. Animal experiments revealed that lung hemorrhage was significantly reduced five days post-infection (dpi) upon administration of 500 mg/kg of NHC, suggesting that NHC is a strong candidate for oral prophylaxis against SARS-CoV replication and related disease [40]. Notably, the study also demonstrated significantly reduced viral titer in the lungs at 48 h after administration of 500 mg/kg NHC [40]. NHC has been postulated to affect the thermodynamics of the secondary structure of RdRp in SARS-CoV, leading to the inhibition of viral replication [42]. Furthermore, treatment with 6 µM of NHC yielded a 90% viral reduction in SARS-CoV-infected Vero 76 cells—confirmed by cytopathic effect inhibition and neutral red uptake assays [42]. These results illustrate that Molnupiravir is a strong candidate for inducing RNA mutagenesis in viruses, thereby reducing and arresting replication capacity of SARS-CoV and SARS-CoV-2. 

## 3. The General Genomic Organization of SARS-CoV-2

**Figure 1:**
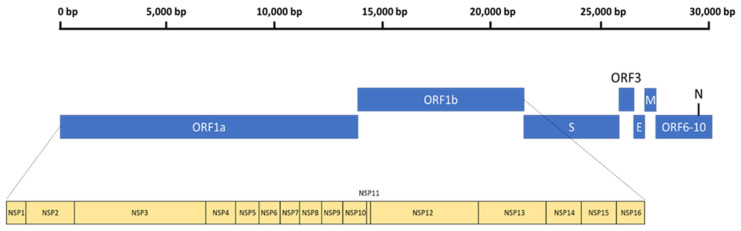
The general genomic organization of SARS-CoV-2.

The general genomic configuration of SARS-CoV-2 closely resembles its beta-coronavirus counterparts, SARS-CoV and MERS-CoV [43]. SARS-CoV-2 is an enveloped, non-segmented, positive-sense RNA virus with a size of 65–125 nm in diameter [44]. Within the envelope resides a 29.9-kb RNA genome, with two-thirds containing the open reading frame 1a and 1b (ORF1ab) replicase that also encodes various non-structural proteins (NSP1–16) [45]. The remaining one-third encodes different structural proteins, namely the spike (S), envelope (E), membrane (M), and nucleocapsid (N) proteins [45]. In addition, there are multiple ORFs at the 3′-end, which encode several accessory proteins (Figure 1). The typical organization of the SARS-CoV-2 genome can be denoted as: 5′-leader-UTR-replicase-S-E-M-N-3′-UTR-poly(A) tail—with the accessory genes scattered between the structural genes (S-E-M-N) at the 3′ end [46].

**Figure 2:**
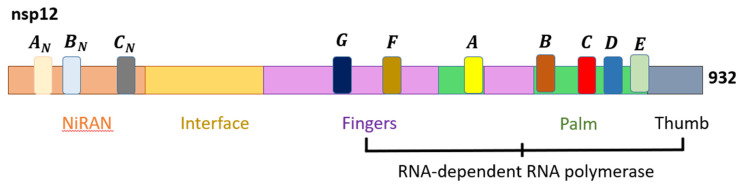
The domain structure of SARS-CoV-2 NSP12 that comprises the nidovirus RdRp-associated nucleotidyltransferase (NiRAN), interface, and RdRp domains [47].

While the structural proteins play important roles in the integrity of new virions, the NSPs are vital for viral replication. Thus, NSP3 and NSP5 impede innate immunity, causing aberrant cytokine expression and viral polyprotein cleavages [47]. Notably, NSP12 is critical for viral replication, and the RdRp complex (RNA replicase) plays a vital role in genome replication and viral transcription [48]. The positive-ssRNA genome of SARS-CoV-2 can function as mRNA for direct protein translation, or serve as template for the production of the negative-strand RNA via RdRp [49]. The SARS-CoV-2 RdRp structure is a complex that comprises NSP12, NSP7, and NSP8, similar to SARS-CoV. Within the NSP12 catalytic subunits are three right-handed structures, namely the fingers (residues 366–581 and 621–679), palm (residues 582–620 and 680–815), and thumb (residues 816–920) subdomains, with polymerase motifs A to G spanning across the RdRp domain (Figure 2). NSP7 and NSP8 are predicted to further stabilize the conformation of NSP12, thereby enhancing the binding and processivity of the RdRp complex [50]. The general viral replication process initiates via nucleotide triphosphate (NTP) binding. This is then followed by a conformational change of the active site, leading to phosphatidyl transfer and subsequent formation of the phosphodiester bond with the existing nucleotide chain. This process is aided by magnesium (Mg^2+^) ions. Finally, the translocation of the newly bound NTP and chain elongation occur [51].

Given the highly conserved nature of RdRP within the coronavirus family, RdRp constitutes an attractive target for developing drug therapies against COVID-19 [16]. With the success of other antiviral interventions against RdRp (such as Remdesivir), it is pertinent to elucidate the mechanisms of Molnupiravir, an N4-hydroxycytidine that inhibits SARS-CoV-2 viral replication.

## 4. The Molecular Mechanisms of Molnupiravir on SARS-CoV-2

As mentioned, the RdRp is well-conserved among coronaviruses, in which it plays a pivotal role in the replication of the SARS-CoV-2 genome. RdRp catalyzes viral RNA replication from the original template, where RdRp synthesizes the complementary negative-sense RNA genome (minus-gRNA) from the positive-strand template. The minus-gRNA serves as a new template for further replication of the positive-sense RNA genome or plus-gRNA [52]. Hence, RdRp is a promising target for treatment strategies against COVID-19. To date, there are a limited number of FDA-approved RdRp inhibitors against SARS-CoV-2, including Remdesivir [53].

The main mechanism underpinning NHC is the inhibition of RdRp by acting as a ribonucleoside analog for RNA polymerase. Unlike Remdesivir which attenuates RNA synthesis, NHC primarily functions as a mutagen by increasing the frequency of transition mutations (G-to-A and C-to-U) [25,54]. A two-step model is deduced for NHC-induced RNA mutagenesis. As NHC enters the cell, it is cleaved in the plasma, followed by phosphorylation by host kinases into its active form, NHC-5′-triphosphate (NHC-TP) [55]. Subsequently, NHC-TP is incorporated into the synthesized minus-gRNA and sub-genomic RNA by RdRp, instead of C or U (when referring to the plus-gRNA template). Notably, NHC-TP predominantly competes with C for incorporation as compared to U. As a result, the NHC-TP-containing minus-gRNA is used as a template for the synthesis of plus-gRNA and positive-strand sub-genomic mRNA, culminating in mutations in the positive-strand RNA products and the formation of non-functional viruses [56]. 

**Figure 3:**
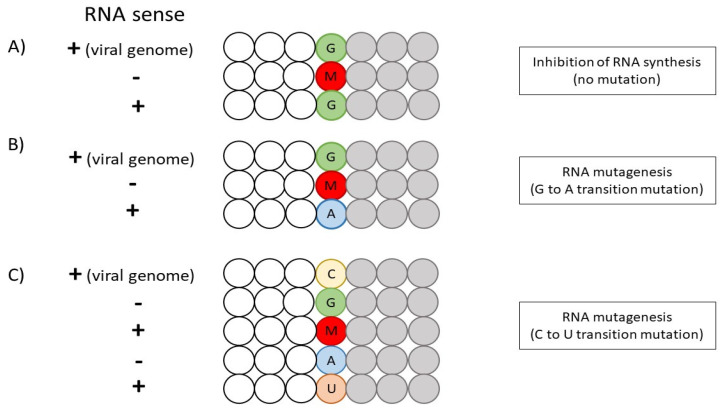
An overview of NHC-induced mutagenesis of SARS-CoV-2 RNA. (**A**) The inhibition of RNA synthesis via [G: NHC-TP: G] base-pairing. (**B**) NHC-induced RNA mutagenesis via G-A transition mutation. (**C**) NHC-induced RNA mutagenesis via C-U transition mutation. The letters G (green), C (yellow), A (blue) and U (orange) represent the ribonucleotide bases, while the letter M (red) refers to Molnupiravir (NHC-TP). The gray circles illustrate the incorporation of NTP after M (NHC-TP).

With the incorporation of NHC-TP in the template, NHC-TP can form a base-pair with either G or A, since NHC exists as two tautomers. The amino or hydroxylamine form of NHC mimics C to base-pair with G via three hydrogen bonds, while the imino or oxime form mimics U to base-pair with A via two hydrogen bonds [57,58]. Although the incorporation of G (NHC-TP: G) in the product-RNA strand will hinder RNA synthesis, the increase in G concentrations will overcome this inhibitory effect with no mutation formed. In contrast, the incorporation of A will result in G-to-A transition mutation due to the [G: NHC-TP: A] base-pairing. The higher intracellular concentrations of A than G may also explain the higher frequency of [G: NHC-TP: A] formation [58]. Furthermore, the presence of C in the positive-strand viral RNA template will result in C-to-U transition mutation due to [C: G: NHC-TP: A: U] base-pairings (Figure 3). Thus, tautomerization and the resultant preference of NHC-TP to function as a C analog promotes the formation of [G: NHC-TP: A] and [C: G: NHC-TP: A: U] base-pairings, which are necessary for NHC-induced mutagenesis [59]. Other than inducing mutagenesis, studies suggest that NHC may disrupt the secondary structure of viral RNA or hinder the release of virions [55,60]. Akin to Remdesivir, NHC possesses the ability to escape viral RNA proofreading by viral exonuclease due to the stability of [NHC-TP: G] and [NHC-TP: A] base-pairings, in which the backtracking of RdRp is not induced [58,61]. The lack of interruption in RNA synthesis may bypass the proofreading ability as well [62].

## 5. Clinical and Preclinical Studies on the Efficacy of Molnupiravir 

Since the discovery of SARS-CoV-2 [63], there have been a few studies on the efficacy and safety of NHC. Based on a randomized, double-blinded Phase I clinical trial on safety, tolerability, and pharmacokinetics, NHC illustrated good potential for SARS-CoV-2 inhibition [64]. The participants (18–60 years old, predominantly Caucasian males with a mean body mass index of 24.4–25.4 kg/m^2^) were subjected to either single-ascending dosage (50–1600 mg NHC or placebo) or multiple-ascending dosage (50–800 mg NHC or placebo, b.i.d.) for 5.5 days. The NHC pharmacokinetic profile revealed relatively good plasma absorption and safety across doses ranging from 50 to 1600 mg. The study also suggested that food intake does not influence the therapeutic efficacy of NHC. Overall, the reported adverse effects for single-dose and multiple-dose NHC include headache (12.5%), diarrhea (7.1%), and rashes. There were minimal negative effects on vital functions, electrocardiogram data, and hematological parameters, i.e., NHC is generally safe within the stipulated dosage and study timeframe. 

Interim data from a phase III clinical trial of 775 patients released by Merck Sharp and Dohme (MSD) concluded that NHC is capable of reducing the risk of hospital admission and death by 50%. The data reported that 7.3% (28 of 385) of NHC-treated patients were admitted to the hospital or died as compared to 14.1% (53 of 377) of placebo-treated patients over the course of 29 days. No further deaths were reported in NHC-treated groups after day 29, while eight deaths were observed in the placebo-treated group. Although the incidence of adverse events was similar in both groups (35% NHC versus 40% placebo), there were fewer participants from the NHC group who discontinued treatment due to adverse events from the placebo group (1.3% versus 3.4%) [65,66]. 

The antiviral activity of NHC has also been demonstrated by in vivo and in vitro preclinical experiments. For instance, the numbers of infectious particles were successfully reduced by 4.4 logs (at 24 h) and 1.5 logs (at 48 h) in NHC-treated immunodeficient mice with implanted human lung tissue [67]. NHC administration at 12 h prior to SARS-CoV-2 infection, and at every 12 h thereafter, reduced virus titers by 100,000-fold. These studies suggest that NHC clearly exerts a prophylactic effect, and can be a strong candidate to treat SARS-CoV-2 infection, although early administration is preferred for superior outcomes [67]. Similarly, Cox et al. (2021) documented a significant reduction of virus shedding at 12 h and 36 h (peak shedding) post-infection in NHC-treated ferret models (fed b.i.d.). Infectious particles were undetectable within 24 h of treatment, with only traces of SARS-CoV-2 RNA detected in nasal tissues. Notably, the close proximity of NHC-treated infected ferrets with two untreated ferrets for 3 days revealed no infectious particles and SARS-CoV-2 RNA in the nasal lavages and intestinal tissue samples from the untreated contacts, further suggesting the efficacy of NHC to inhibit SARS-CoV-2 replication [68]. 

Furthermore, one study showed that 10 μM of NHC successfully inhibited virus production in primary human airway epithelial cells with a maximal titer reduction of >5 logs of MERS-CoV and >3 logs of SARS-CoV. Upon NHC administration at 10 μM dosage, the mutation rate of NHC-treated MERS-CoV RNA was significantly elevated by up to ten-fold. With NHC treatment, potent dose-dependent virus reduction was observed, i.e., 1.5 × 10^2^ PFU/mL with 10 μM NHC versus 2.96 × 10⁴ PFU/mL with 1 μM NHC, compared with 3.96 × 10⁶ PFU/mL for vehicle control. Overall, error rates of 3-fold and 6-fold were accompanied by 138-fold and 26,000-fold reductions in virus titer during treatment with 1 μM and 10 μM of NHC, respectively [40]. Additionally, a significant decrease in lung hemorrhage was also observed in infected mice administered with 500 mg/kg of NHC [40]. Collectively, these experiments suggest that NHC confers a prophylactic effect, and that earlier administration of NHC could significantly reduce SARS-CoV-2 replication and lung hemorrhage, thereby improving the survival rate of COVID-19 patients. There are multiple clinical trials (NCT04575584, NCT04939428, NCT04405739, NCT04746183) to evaluate the efficacy and safety of NHC against SARS-CoV-2 infection [69,70,71,72]. 

Despite the encouraging outcomes, the mutagenic effect of NHC has raised concerns on its potential host cell mutagenesis. One study demonstrated that there is no accumulation of host ISG15 mutations at high NHC concentrations (up to 500 mg/kg), accompanied by efficient removal of ribonucleotides from host DNA [40,73]. Mutagenicity assays conducted on high concentrations of NHC displayed no significant differences in mutation rates between NHC-treated and untreated animals. NHC was also shown to be safe from inducing chromosomal damage in micronucleus in vitro and in vivo [74]. There was also no increase in mutational load in SARS-CoV-2-infected and NHC-treated golden hamster lung biopsies [75]. Nevertheless, there are contradictory reports that NHC may potentially cause mutagenesis in human host cells. One report stated that NHC could be metabolized to 2′-deoxyribonucleotide form (dNHC) by host ribonucleotide reductase, leading to the incorporation of DNA and subsequent host mutagenesis. The same study also illustrated that the ribonucleoside form of NHC (rNHC) was mutagenic to Chinese hamster ovary (CHO-K1) cells by converting to dNHC in a dose-dependent manner, resulting in the loss of HPRT gene function via missense substitutions and frameshift mutations. This phenomenon was further supported by rNHC-treated cells which induced resistance to the toxic base analog 6-TG (which functional HPRT genes are sensitive to) at concentrations that do not affect cell viability [76]. Animal reproduction studies have also alluded that Molnupiravir may cause fetal harm when administered to pregnant women. Hence, the drug is not recommended for use during pregnancy [77]. Despite the limited reports on host cell mutagenesis, future detailed studies and long-term observations are essential for patients administered with NHC to further evaluate its safety profile in the longer term.

## 6. Other Emerging Antiviral Drugs against COVID-19

To date, several drugs have undergone clinical trials or are being evaluated for the treatment of COVID-19. Ivermectin (IVM) and Remdesivir have attracted attention and some controversy on their use for COVID-19 treatment. Ivermectin is an FDA-approved anti-parasitic drug for the prevention of heartworm disease in certain animals such as cattle, dogs, and horses. Studies have shown the efficacy of IVM against viruses such as Zika virus and IAV [78,79]. IVM is proposed to disrupt the host importin heterodimer complex (IMPα/β1) for the transport of SARS-CoV-2 proteins such as NSP12-RdRp during infection, thereby hijacking viral replication [80,81]. Of note, IMPα/β1 is a vital complex that facilitates the transport of host proteins such as STAT proteins for normal cellular activities [80,82]. In addition, IVM is capable of inhibiting SARS-CoV-2 viral entry that requires the attachment of viral S protein to the host ACE2 receptor. This is achieved via the altered interaction between the S protein receptor-binding domain (RBD) and host ACE2, allowing a conformational change of the S protein that subsequently obstructs viral entry and hampers viral replication [83]. Furthermore, the IVM carbonyl group is thought to form hydrogen bonds with the active site residues (Cys145 and His41) of the 3CLpro monomer, an important viral protease required to generate vital proteins for replication, such as NSP12-RdRp [84]. This leads to destabilization and dysfunction of the 3CLpro complex, thereby restricting viral replicative capacity [85]. However, some studies have indicated the lack of significant positive outcomes between IVM-treated patients and control patients [86,87,88]. Moreover, IVM is associated with multiple side-effects, including tremors, ataxia, nausea, headaches, tachycardia, coma, and even death [89]. 

Remdesivir is an FDA-approved antiviral drug for the treatment of COVID-19 by targeting viral RdRp. Upon the entry of Remdesivir into the host cell, the monophosphoramidate nucleoside prodrug is phosphorylated into active Remdesivir triphosphate (Remdesivir-TP). Similar to NHC, Remdesivir-TP competes with ATP for incorporation by RdRp into the RNA genome. As such, the activity of RdRp is abrogated after a three-nucleotide elongation, in which the steric hindrance between Ser861 and 1′-CN group of Remdesivir attenuates the translocation of RdRp into the fourth position, resulting in delayed chain termination and subsequent inhibition of viral replication [90]. Although the attenuation of RNA synthesis is deemed to be the primary mechanism of Remdesivir, higher concentrations of NTP can reduce the efficacy of chain termination. For instance, 10 μM concentration of NTP can cause up to 90% read-through, and the production of full-length products [91]. Consequently, the lack of chain termination allows Remdesivir to be incorporated into the initial RNA copies, serving as a template for further RNA replication (template-dependent inhibition mechanism) [92]. Hence, the viral RNA copies generated from the Remdesivir-TP-embedded template will not be fully functional. Despite the positive outcomes of Remdesivir, its associated adverse effects have raised some concern among clinicians and the public [93]. The administration route of Remdesivir is limited to intravenous delivery which limits its usage on larger scales.

In comparison, NHC is associated with relatively few side-effects such as headache and diarrhea. The oral route of NHC also allows infected patients to readily access COVID-19 medication, thus mitigating crowding in hospitals. The prophylactic use of NHC is beneficial especially for regions with low COVID-19 vaccination rates. Furthermore, NHC is effective against Remdesivir-resistant viruses and against several other coronaviruses (HKU3, HKU5, and SHC014), suggesting that NHC is not restricted by the variation in amino acid sequence of RdRp, and possesses efficacy against a broader range of coronaviruses [40].

## 7. Conclusions

The rapid progression and sustained global persistence of COVID-19 has placed enormous pressure to develop and harness therapeutic agents against SARS-CoV-2. In such critical times, drug repositioning can play a pivotal role to fulfil this urgent need. Molnupiravir (NHC) possesses antiviral activity against RNA viruses such as IAV, VEEV, Ebola virus and SARS-CoV, and shows promise as an antiviral drug against SARS-CoV-2. The main mode of antiviral action of Molnupiravir involves the inhibition of RdRp by acting as a ribonucleoside analog for viral RNA polymerase. NHC mainly functions as a mutagen by increasing the frequency of transition mutations (G-to-A and C-to-U) in the viral genes. As a consequence, the negative-gRNA strand containing NHC-TP leads to mutations in the complementary positive-strand RNA, thereby generating non-functional viruses. Owing to its antiviral and prophylactic effects, Molnupiravir may be deployed as one therapeutic solution to ameliorate SARS-CoV-2 infection, by reducing disease severity and fatality. Finally, more comprehensive clinical trial data and long-term surveillance are urgently warranted to support the clinical utility and safety of Molnupiravir against COVID-19.

## Data Availability

Not applicable.
